# Correlation between radial peripapillary vascular density and reduced central corneal thickness in glaucoma suspect patients

**DOI:** 10.1186/s12886-022-02628-z

**Published:** 2022-10-31

**Authors:** Ayman Lotfy, Hala Kamal Mattout, Sameh Mosaad Fouda, Sahar Hemeda

**Affiliations:** Zagazig University, Zagazig, Egypt

**Keywords:** glaucoma suspect, Peripapillary vascular network, Retinal fiber layer, Corneal thickness

## Abstract

**Background:**

Optical coherence tomography (OCT) angiography (OCTA) provides a quantitative assessment of the microcirculation of the retina and choroid. It may precede the retinal nerve layer (RNFL) and optic disc head defects. Retinal nerve fiber layer defects and reduced central corneal thickness (CCT) are important parameters in the assessment of a glaucoma suspect patients. The aim of this study is to investigate any possible relationship between the reduced central corneal thickness and the radial peripapillary capillary (RPC) density defect in glaucoma suspect.

**Methods:**

In this cross sectional study, 92 eyes were incorporated. Peripapillary OCT angiography (4.5 mm) and Anterior segment OCT for corneal pachymetry were done. C/D Ratio, thickness of nerve fiber layer, the blood flow indices and central corneal thickness also were evaluated.

**Results:**

In eyes of glaucoma suspect patients; a significant positive correlation between CCT and total RPC density was detected (r = 0.38, P < 0.001). A strong positive correlation was also found between total RPC and peripapillary RNFL thickness (r = 0.55, P < 0.001).

**Conclusion:**

Reduced central corneal thickness and peripapillary capillary density are two screening parameters for glaucoma suspect patients. The radial peripapillary capillary density is a valid diagnostic tool for glaucoma.

**Supplementary Information:**

The online version contains supplementary material available at 10.1186/s12886-022-02628-z.

## Introduction

Glaucoma is an optic neuropathy characterized by progressive damage of retinal ganglion cells. The factors of its progression have not been completely known. Glaucoma causes gradual and progressive visual field loss and irreversible blindness with 10% being bilaterally blind.

A glaucoma suspect identified during routine screening is a person who has one or more risk factors which increase the possibility of developing glaucoma in the future [1–3]. The risk factors include increased intraocular pressure, abnormal optic disc, visual fields defects, retinal nerve fiber layers defect, abnormal angles and positive family history of glaucoma [4]. Structural and vascular theories are the main theories of pathogenesis of glaucoma [4, 5]. Neural damage and focal defect in the lamina cribrosa (LC) have been reported to be the most significant structural changes of glaucoma associated with visual field (VF) and retinal nerve fiber layer (RNFL) loss [6, 7].

Vascular changes are preceding or coinciding with the onset of glaucoma and its progression [8–12]. Some studies reported that these changes occur before retinal nerve fiber layer (RNFL) thinning and visual field defect, so it is important to evaluate the blood perfusion at the site of the optic disc during screening persons with risk of developing glaucoma [13, 14]. Optical coherence tomography angiography is a more advanced noninvasive technique for imaging and detecting blood flow through the motion contrast generated by red blood cells. It provides quantitative data for assessment of the microcirculation of the retina and choroid among different levels. This advanced technology gives new information about the pathophysiology of glaucoma for early diagnosis and management [15]. Central corneal thickness (CCT) is another important parameter in the assessment of any glaucoma suspect patient. There are different factors that may affect the CCT including: ethnicity, genetics, age, glaucoma treatment and previous refractive surgery; these factors need to be taken into consideration when evaluating CCT of a glaucoma patient and its effect on interpretation of intraocular pressure (IOP) levels [16–19].

Glaucoma suspect patients with thin cornea and reduced peripapillary vascular density have an increased possibility of developing glaucoma and visual field defects. The aim of this study is to investigate any possible relationship between the reduced central corneal thickness and the radial peripapillary capillary density defect in glaucoma suspects.

## Methods

This cross sectional observational study was composed of patients who were diagnosed as glaucoma suspect. The institutional review board approved the study (Ethical approval: 717–13, Zagazig university). The Declaration of Helsinki tenets were followed. Written informed consent was obtained before enrolment of patients. Patients were collected from ophthalmology outpatients’ clinic of Zagazig University. The study also included patients’ data collected from November 2019 to November 2021 at the Alpha Eye Hospital, Zagazig Egypt. The charts of patients who had CCT measured by the Optovue AS-OCT, RNFL thickness and radial peripapillary capillary density scanned by Optovue OCT-Angio were reviewed for the study.

The inclusion criteria were those patients who presented with normal intraocular pressure (IOP) and with a vertical C/D ratio ≥ 0.5 or asymmetric optic discs in both eyes (asymmetry of C/D ratio between two eyes ≥ 0.2 that was not caused by the difference in optic disc size or shape).

**The exclusion criteria** were previous refractive surgery, corneal scarring, keratoconus, high intraocular pressure (more than 22 mm Hg), history of anti-glaucoma therapy, previous glaucoma surgery, trauma, pathologic myopia, diabetic retinopathy, vascular occlusion, uveitis and patients with RNFL thickness outside normal limits.

After taking their informed consents, all the subjects were asked for detailed ocular and systemic histories and they underwent complete ophthalmic examination including the following: best corrected visual acuity (BCVA), anterior segment examination by slit-lamp bio-microscopy as well as intraocular pressure measurement (IOP) and indirect ophthalmoscopy fundus examination. After examination, all patients had Optical Coherence Tomography (OCT) scanning using the spectral domain OCT system (RTVue OCT; Optovue Inc., Fremont, California, USA). The Optovue system was used to obtain results regarding peripapillary retinal nerve fibre layer (RNFL) thickness, radial peripapillary capillary density (RPC) by OCT-Angiography on optic nerve head and central corneal thickness by anterior segment OCT system; all were taken for every patient at the same setting.

The peripapillary RNFL thickness was measured using 12 radial scans of 3.4 mm in length centered on the optic disc. The GCC scan was also done with a square grid centered on the macula. The built-in software of the Optovue OCT uses the normative database to define cut-offs depending on the statistical distribution of normal eyes. The obtained result for every patient is compared to this normative database and when the values lie outside the normal range, this result is flagged as outside normal limits. The cut-offs used by this normative database are typically the lower 5% of the normal distribution and they are called ‘borderline’ while the bottom 1% are classified as ‘outside normal limits’. Only patients who were classified as normal or borderline were included in the study.

The corneal thickness was measured by cross sectional corneal images and automated algorithm that detects the distance between the anterior and posterior boundaries by using anterior segment OCT. Images were taken using the “pachymetry” scan pattern settings in the OCT and the cornea was mapped accordingly.

OCT angiography scans were obtained by the spectral domain system (Optovue Inc., Fremont, CA, USA, software version 2017, 1, 0, 151). It has an A-scan rate of 70 kHz per second, using a light source of 840 nm wavelength and a bandwidth of 45 nm. By using motion contrast, the software algorithm compares the consecutive B-scans at the same site to detect the flow. The scan was centered on optic disc (4.5×4.5 mm). Three slab levels; retina slab, radial peripapillary capillary (RPC) slab, and choroid slab. The Angiovue software gave numerical indices of vascular density. Vessel density was defined as the percentage peripapillary area that is occupied by the microvasculature and large vessels. It measured superior, nasal, inferior, temporal, superior hemi and inferior hemi peripapillary regions

### Statistical analysis

Quantitative variables were evaluated by Shapiro–Wilk test to identify their distribution characteristics. Normally distributed variables were described using mean (standard deviation (SD)), while non-normally distributed data were described using median (interquartile range (IQR)). Pearson correlation coefficient was used to study the association between the continuous variables. To evaluate the capacity of RPC to distinguish between patients with normal RNFL thickness and those with borderline RNFL thickness, receiver operating characteristics curve (ROC) was constructed and the area under the curve (AUC) was estimated. A P value of ˂ 0.05 was considered significant. Data analysis was performed using Statistical Package of Social Services, version 25 (SPSS) (IBM, 2017)

## Results

**Fig. 1:**
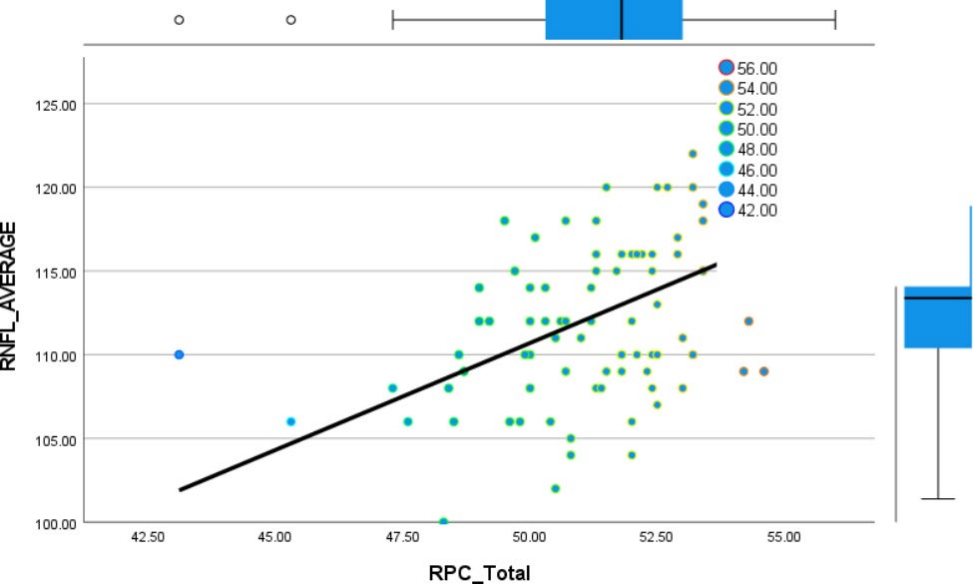
Shows the relation between both total RPC and the average peripapillary RNFL thickness:

**Fig. 2:**
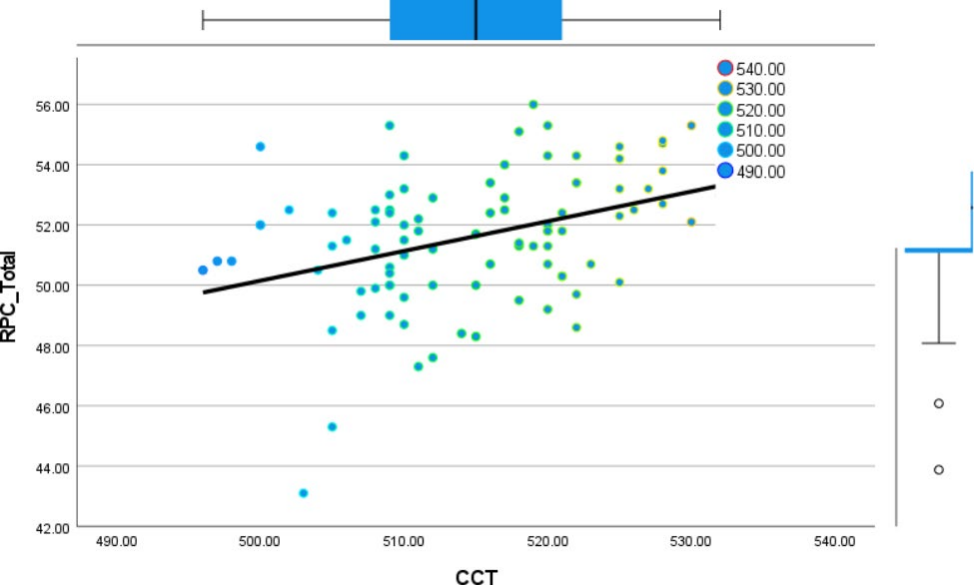
The relation between both CCT and total RPC:

**Fig. 3:**
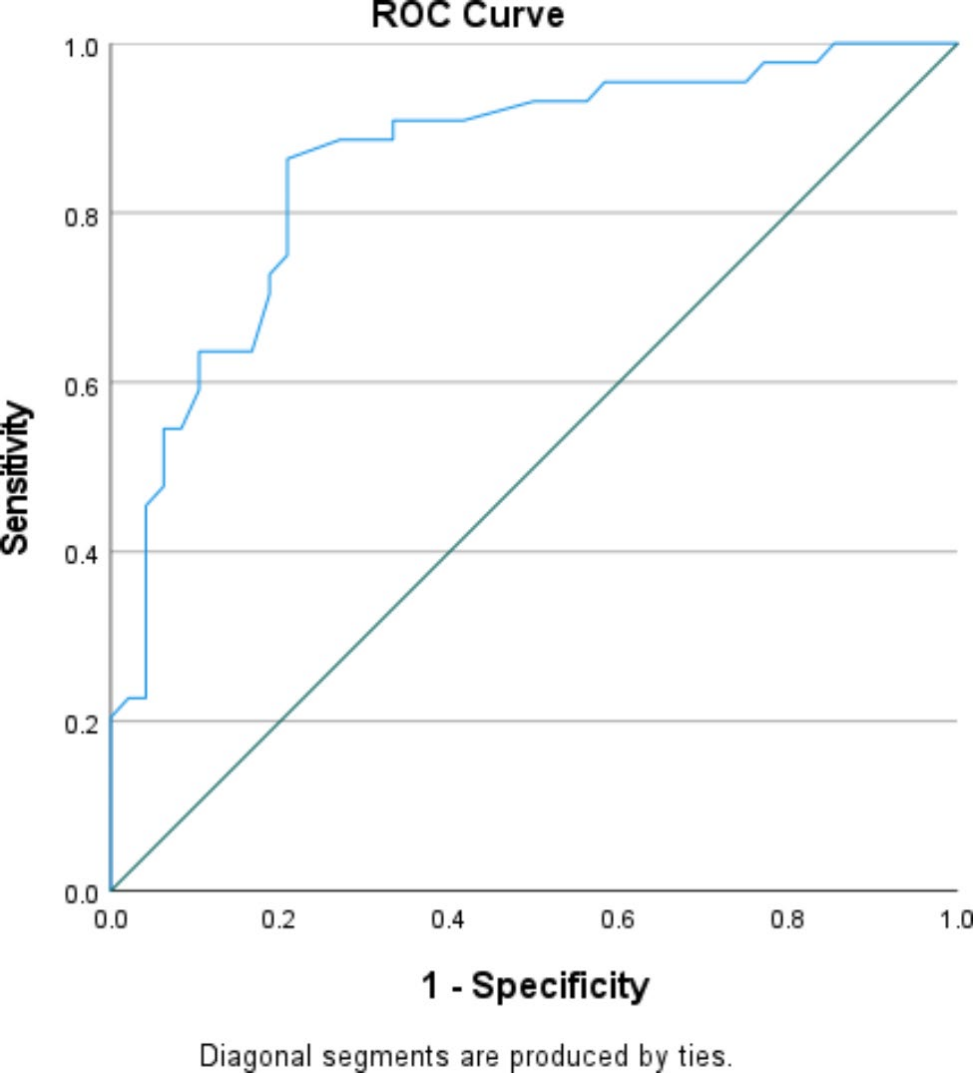
The ROC curve for the total RPC density

A total of 46 patients (92 eyes) fulfilling the inclusion criteria were included for data analysis, the mean age of the studied patients was 46.8 ± 9.1 years, they were distributed as 30 females and 16 male patients. Demographic and clinical data of the studied patients are presented in Table 1. The correlation between total RPC, hemi superior, hemi- inferior RPC and the average RNFL, superior and inferior RNFL is presented in Table 2. There was a significant positive correlation between the total RPC and the average peripapillary RNFL thickness. Also, a significant positive correlation was found between hemi-superior RPC, hemi-inferior RPC and the superior RNFL, inferior RNFL thickness respectively. Figure 1 shows the linear relation between both total RPC and the average peripapillary RNFL thickness. Table 3 shows the significant strong positive correlation found between CCT and the RNFL (total, superior and inferior). This linear relation is presented in the scatter plot shown in Figure 2. Table 4 shows the correlation between RPC and central corneal thickness, C/D ratio and IOP. Significant positive correlation was detected only between CCT and total RPC as well as both hemi-superior RPC and hemi-inferior RPC. Negative weak correlation was depicted between RPC and both IOP and C/D ratio but it was not statistically significant. There was a stronger positive correlation between CCT and RPC in eyes with borderline RNFL thickness than in those with normal RNFL thickness, but the correlation was statistically significant in both of them. This difference is presented in Table 5. Figure 3 shows the ROC curve constructed to evaluate the ability of RPC density to distinguish between patients with normal RNFL thickness and those with borderline RNFL thickness; the AUC was 0.858 indicating that RPC density has a diagnostic accuracy. AUC for the ROC curve constructed for both hemi-superior RPC and hemi-inferior RPC density was 0.910 and 0.839 respectively depicting the higher diagnostic accuracy of the hemi-superior RPC density. Table 6 describes the performance of the total RPC as a predictor of glaucoma in the studied group. The cutoff point of total RPC equals to or less than 51.4 and it can be used as a predictor for presence of glaucoma with sensitivity of 72.7%, specificity of 81.2%, PVP of 66.7% and PVN of 85.7%.

**Table 1:** Demographic and some clinical data of the studied patients:

Age (years) Mean (SD)	46.8 ± 9.1
**Gender** Male Female	16 (35%) 30 (65%)
**IOP (mmHg)** Mean (SD)	18.1 ± 0.7
**Cup/Disc Area Ratio** Mean (SD)	0.61 ± 0.08
**Central corneal thickness(microns)** Mean (SD)	512.2 ± 8.69

**Table 2:** Mean RNFL and RPC (average, superior and inferior) and the correlation between them:

	RNFL(microns)	RPC(percentage)	r	P
**Average or Total**	112.75 ± 5.17	51.61 ± 2.22	0.55	< 0.001*
**Superior**	116.57 ± 4.5	51.99 ± 2.2	0.4	< 0.001*
**Inferior**	109.15 ± 5.49	49.69 ± 2.3	0.49	< 0.001*

**Table 3:** Correlation between average RNFL thickness and the central corneal thickness

	CCT	
	r	p
**Average RNFL**	0.63	< 0.001*
**Superior RNFL**	0.59	< 0.001*
**Inferior RNFL**	0.56	< 0.001*

**Table 4:** Correlation between RPC and the central corneal thickness, IOP and C/D ratio

	Borderline RNFL		Normal RNFL	
	N = 44		N = 48	
	CCT		CCT	
	r	p	r	P
**Total RPC**	0.6	< 0.001*	0.3	0.002*
**Hemi Superior**	0.5	< 0.001*	0.3	0.008*
**Hemi Inferior**	0.7	0.004*	0.3	0.01*
**Inside disc**	0.07	0.6	0.05	0.7

**Table 5:** Correlation between RPC and the central corneal thickness in eyes with normal and borderline RNFL thickness

	CCT		IOP	
	r	p	r	P
**Total RPC**	0.38	< 0.001*	-0.07	0.47
**Hemi Superior**	0.24	0.02*	-0. 1	0.34
**Hemi Inferior**	0.23	0.03*	-0.17	0.1
**Inside disc**	-0.05	0.61	-0.07	0.48

**Table 6:** Performance of total RPC as a predictor of glaucoma in the studied group:

Variable	Cutoff point	AUC	Sensitivity	Specificity	PVP	PVN	accuracy	P value
RPC total	≤ 51.4	0.858	72.7%	81.2%	66.7%	85.7%	78.3%	< 0.001*

AUC = area under the curve.,PVP=,predictive value positive PVN = predictive value negative

Table 7 shows the logistic regression analysis of factors predicting glaucoma in the studied group. It was found that that low values of RPC, RNFL and CCT were found to be significant predictors for presence of glaucoma after controlling the confounders as age, gender and intraocular pressure. Patients with low RPC measurement are likely to have glaucoma 5 folds (OR = 5.13, P = 0.01) compared to those with high measurements. As regarding RNFL and CCT, those with low values of RNFL and CCT are 1.15, 1.79 folds more likely to have glaucoma compared to those with high values.

**Table 7:** Logistic regression analysis of factors predicting glaucoma in the studied group

Independent factors	B	S.E.	Wald	O.R (95% C.I)	P-value
**RPC total**	1.636	0.671	5.951	5.13 (1.37 − 19.1)	**0.01**
**RNFL**	1.887	0.814	5.347	1.152 (0.03–0.74)	**0.02**
**CCT**	0.584	0.283	4.255	1.79 (1.03–3.12)	**0.03**

OR = odds ratio, CI = confidence interval

**Fig. 4:**
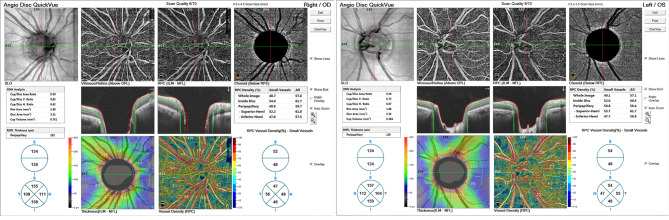
OCTA of glaucoma suspect

**Fig. 5:**
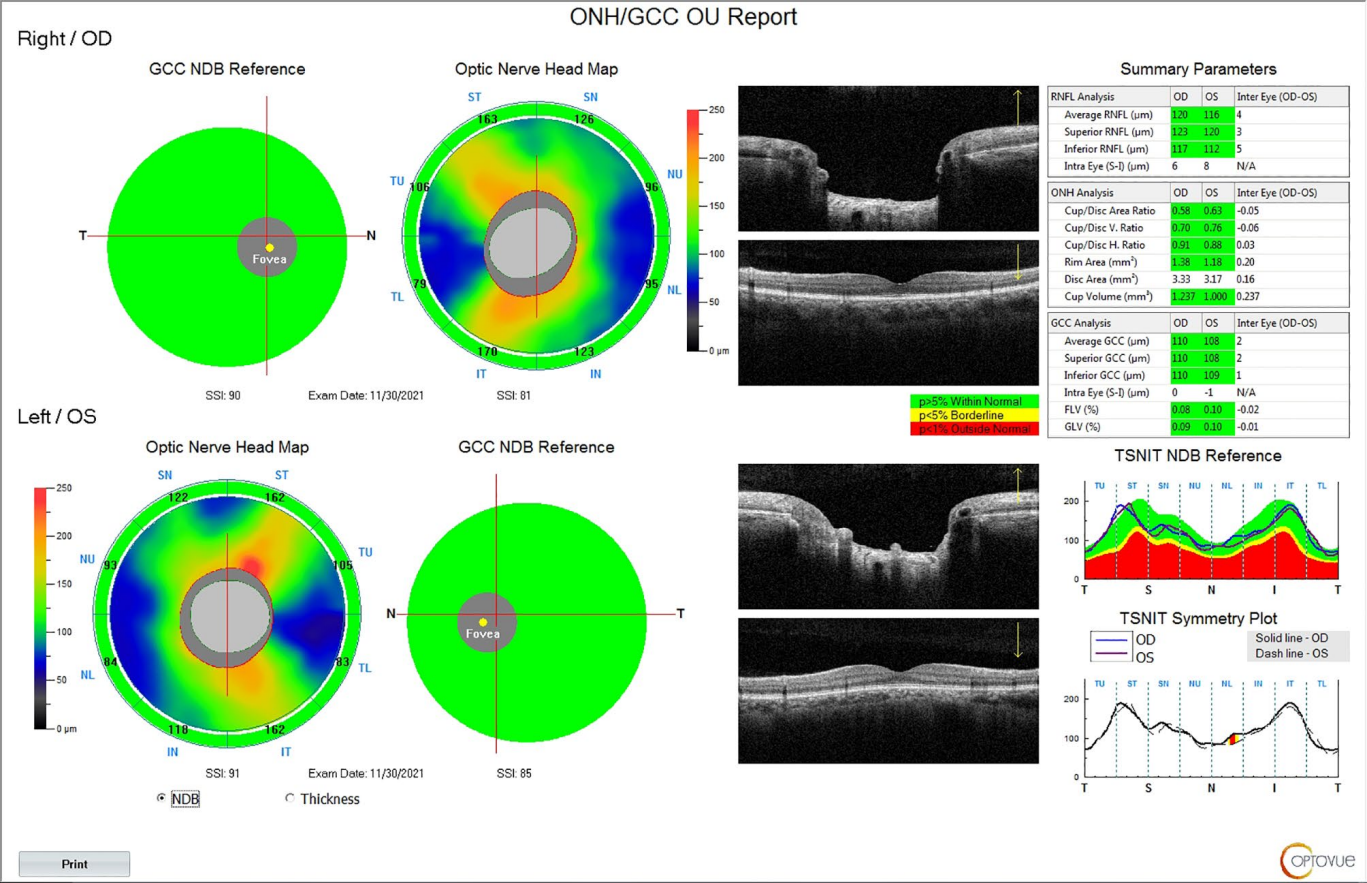
OCT of glaucoma suspect

**Fig. 6:**
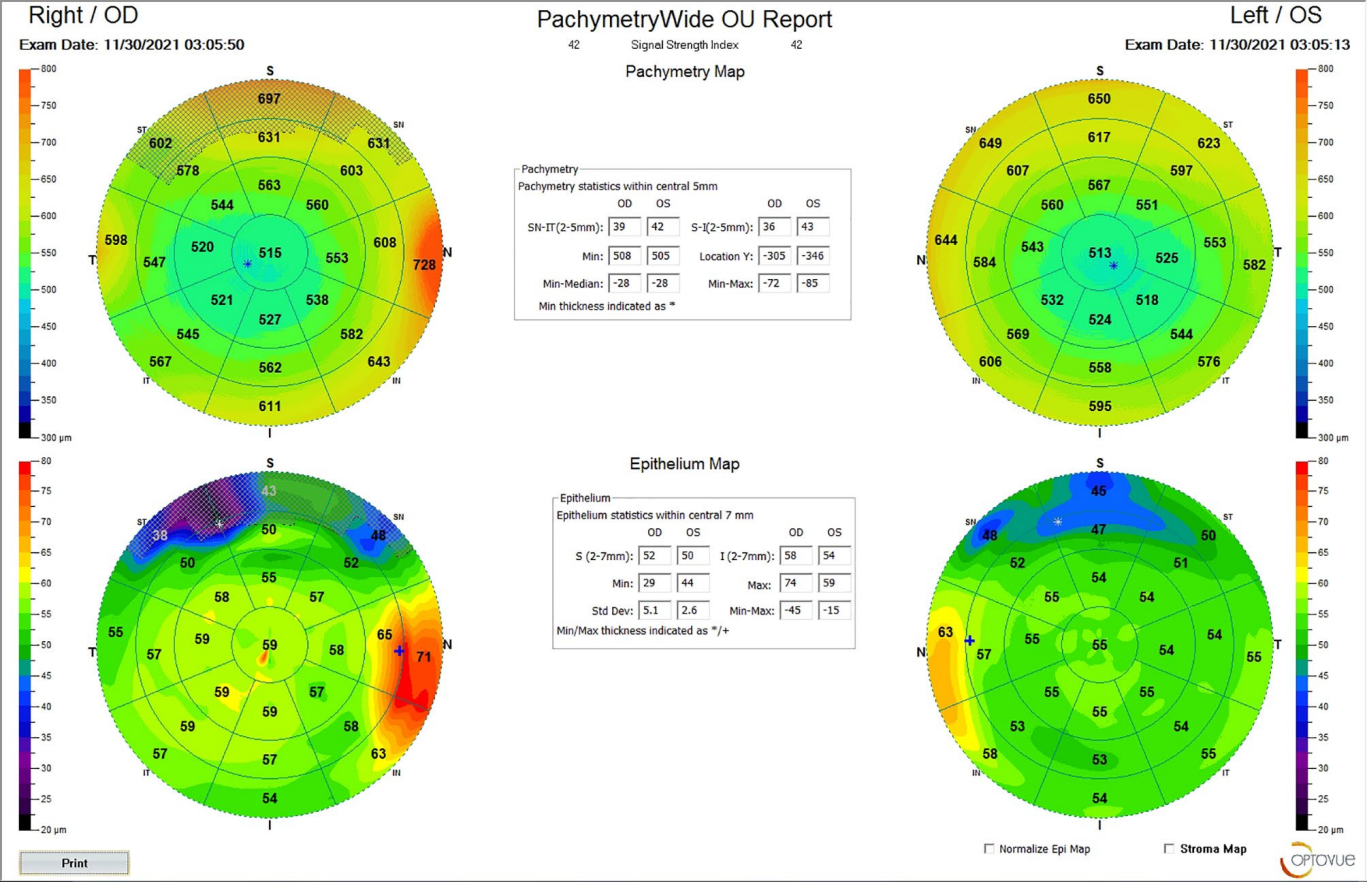
Corneal thickness by OCT of glaucoma suspect

Case study was showed in Figs. 4–6.

## Discussion

Goldman applanation tonometry is considered the gold standard method for intraocular pressure measurement. Applanation tonometer is influenced by several factors. Central corneal thickness remains a factor with a possible impact on the intraocular pressure measurements by applanation tonometry. Reduced corneal thickness is associated with underestimated intraocular pressure [20, 21].

Several studies found that reduced corneal thickness is correlated with progression of glaucomatous optic neuropathy according to the Advanced Glaucoma Intervention Study (AGIS) scale. The reduced central corneal thickness is correlated with visual field defects and optic disc changes. The ocular hypertension treatment study (OHTS) raised the attention towards the reduced corneal thickness and its association with the diagnosis of open angle glaucoma. According to ocular hypertension study, patients with central corneal thickness of less than 555 μm had a greater risk of primary open angle glaucoma than the patients with thickness more than 588 μm. Some studies postulated that reduced central corneal thickness is associated with delayed detection of glaucoma [22, 23].

On the other hand, histopathology studies did not find the relation between thin cornea and thin lamina cribrosa [24].

We found that, there was a significant positive correlation between the total RPC and the average peripapillary RNFL thickness. Also, a significant positive correlation was found between hemi-superior RPC, hemi-inferior RPC and the superior RNFL, inferior RNFL thickness respectively. A significant positive correlation was detected only between CCT and total RPC, both hemi-superior RPC and hemi-inferior RPC. The AUC was 0.858 indicating that RPC density has a diagnostic accuracy. AUC for the ROC curves constructed for both hemi-superior RPC and hemi-inferior RPC density was 0.910 and 0.839 respectively depicting the higher diagnostic accuracy of the hemi-superior RPC density.

Optical coherence tomography angiography (OCT-A) gives quantitative analysis of the retinal vasculature. Decreased vascular density might have the same sensitivity as the retinal nerve fiber layer RNFL defects in diagnosis of glaucoma [14]. It might have the same prognostic value of RNFL thickness for distinguishing normal from glaucoma suspects and glaucoma patients. Studies proved that peripapillary vascular density was lower in glaucoma than normal and glaucoma suspect [25–29].

According to several studies, the width of vascular defect was found to be associated with the elevated IOP and reduced central corneal thickness. Studies showed correlation between the deep vascular defects and IOP, age, axial length and myopia [30–32]. Ocular hypertension suspect cases showed the highest CCT values. Ocular Hypertension cases showed biomechanical characteristics of high CCT and normal or high corneal hysteresis. In kollia et al. study, they found significant correlation between CCT and RPC density. They found statistically significant low RPC density in OHT eyes with OH in the optic nerve inferior hemi-quadrant. The chaung study found significant thinner cornea in normal-tension glaucoma. They found also, that reduced corneal thickness in NTG correlated with decreased RPC peripapillary vessel density [33–35].

OCTA gives promising results in detection of vascular changes in retina and choroid. It might play an important role in management of glaucoma. Further larger studies are needed to evaluate the correlation and agreement of presence of reduced vascular density with the other risk factors of optic disc changes and loss of ganglion cells in glaucoma.

## Conclusion

Reduced central corneal thickness and peripapillary capillary density are two screening parameters for glaucoma suspect patients. The radial peripapillary capillary density can be a valid diagnostic tool for glaucoma.

## Electronic supplementary material

Below is the link to the electronic supplementary material.

Supplementary Material 1

## Declarations

### Availability of supporting data

the data collected and analysed in this study are available and included in this published article (supplementary file).
